# Single‐cell characteristics and malignancy regulation of alpha‐fetoprotein‐producing gastric cancer

**DOI:** 10.1002/cam4.5883

**Published:** 2023-04-05

**Authors:** Yanxia Mei, Ming Li, Jihang Wen, Xiangxing Kong, Jun Li

**Affiliations:** ^1^ Department of Colorectal Surgery and Oncology, Key Laboratory of Cancer Prevention and Intervention, Ministry of Education, The Second Affiliated Hospital Zhejiang University School of Medicine Hangzhou Zhejiang China; ^2^ Zhejiang University Cancer Center Hangzhou Zhejiang China

**Keywords:** alpha‐fetoprotein, alpha‐fetoprotein‐producing gastric cancer, Dickkopf‐1, gene expression omnibus, malignancy, molecular feature, single‐cell RNA sequencing, the cancer genome atlas

## Abstract

**Objective:**

To characterize alpha‐fetoprotein (AFP)‐producing gastric cancer (AFPGC) at the single‐cell level and to identify regulatory factors for AFP expression and malignancy.

**Methods:**

ScRNA‐seq was performed on two tumors collected from patients with AFPGC. InferCNV and sub‐clustering were applied to identify typical AFPGC cells, followed by AddModuleScore, pathway enrichment, Pseudo‐time, and Scenic analyses. Data from a gastric cancer (GC) cohort were collected for conjoint analysis. The analytical results were verified by cell experiments and immunohistochemistry.

**Results:**

AFPGC cells are similar to hepatocytes in transcriptome and transcriptional regulation, with kinetic malignancy‐related pathways, compared to the common malignant epithelium. In addition, compared to common GC cells, malignancy‐related pathways, such as epithelial‐mesenchymal transition (EMT) and angiogenesis, were upregulated in AFPGC. Mechanistically, Dickkopf‐1 (DKK1) was found to be associated with AFP expression and malignant phenotype upon combining our scRNA‐seq data with a public database, which was further verified by a series of in vitro experiments and immunohistochemistry.

**Conclusion:**

We demonstrated the single‐cell characteristics of AFPGC and that DKK1 facilitates AFP expression and malignancy.

## Graphical abstract

By applying single‐cell transcriptome sequencing to AFP‐producing gastric cancer (AFPGC), our study revealed novel molecular features of this rare disease. We also discovered a key gene, DKK1, that might regulate the malignant phenotype and AFP expression of AFPGC through a joint public database.

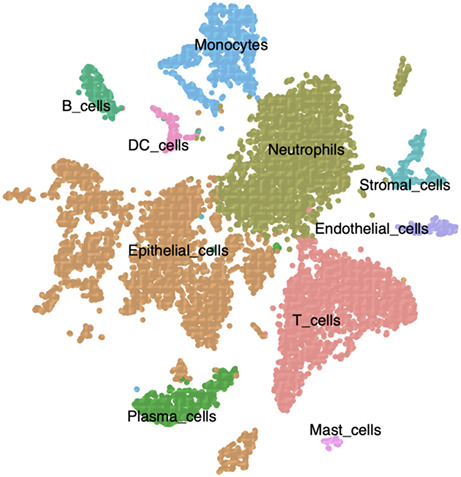

## 1 INTRODUCTION

Alpha‐fetoprotein‐producing gastric cancer (AFPGC) is a rare but highly malignant subtype of gastric cancer (GC) characterized by elevated serum alpha‐fetoprotein (AFP) levels (>20 ng/mL). AFPGC accounts for 1.3%–15% of all GCs.^1^ Patients with AFPGC have an aggressive clinical course, early occurrence of multiple organ metastases, primary multidrug resistance, and a poor prognosis. The 5‐year survival rate of patients with stage IV AFPGC is 4.4%.^2^ The pathogenesis of AFPGC is still not fully understood and effective therapeutic targets are lacking.

In recent years, whole‐exome sequencing (WES) has been used to study the genetic mutation profiles of AFPGC. In our previous study, we performed whole‐exome sequencing of 29 cases of AFP‐producing gastrointestinal cancer (AFPGI), including 26 cases of AFPGC, but no characteristic genetic mutations were found.^3^ Teng et al. performed a similar study on 58 AFPGC tissues and matched normal tissues, identifying amplification of CCNE1 (19q12) and ERBB2 (17q12); the therapeutic value of targeting CCNE1 and ERBB2 was further validated using patient‐derived xenograft (PDX) models.^4^ However, according to a pan‐cancer analysis by Zack et al., the amplification of the two targets is prevalent in many types of cancer^5^; therefore, CCNE1 and ERBB2 may not be characteristic genetic alterations of AFPGC.

Dickkopf‐1 (DKK1) is a repressor of the classical WNT signaling pathway, is physiologically expressed in the bladder and placenta, and is also involved in human embryonic development and bone formation.^6^ Previous studies have shown that DKK1 is aberrantly expressed in various tumor types, such as glioma and liver cancer^7^, ^8^; however, its effect on the malignant phenotype varies among tumors. In hepatocellular carcinoma, DKK1 promotes tumorigenesis, including invasion, metastasis, and angiogenesis,^9^, ^10^, ^11^, ^12^ and is associated with poor prognosis.^13^ In addition, DKK1 has a more significant diagnostic value than AFP in hepatocellular carcinoma.^14^ It has been recently demonstrated that DKK1 promotes immune escape and impairs anti‐PD‐1 therapeutic efficacy in GC.^15^ However, there is no literature on the relationship between DKK1 and the malignant phenotype or AFP expression in AFPGC.

AFPGC is speculated to originate from common adenocarcinoma cells, which show dedifferentiation toward pluripotent embryonic stem cells during tumor progression, undergo lineage reprogramming, and acquire hepatoid differentiation and the ability to secrete AFP.^1^, ^16^, ^17^ Therefore, instead of searching for specific gene alterations, we performed single‐cell transcriptome sequencing of AFPGC to explore the complex cellular components and evolutionary relationships within their tissues and to characterize the molecular profile of AFPGC cells at the single‐cell level. We further combined our single‐cell data with a public database to reveal the relationship between DKK1, the malignant phenotype of AFPGC, and the regulation of AFP expression, which were confirmed by immunohistochemistry and in vitro experiments.

## 2 MATERIALS AND METHODS

### 2.1 Tissue preparation and single‐cell RNA sequencing of AFPGC

#### 2.1.1 Tissue dissociation and preparation of single‐cell suspensions

Two tumor tissues were obtained from patients newly diagnosed with AFPGC at The Second Affiliated Hospital, Zhejiang University School of Medicine, Hangzhou. The tissue was placed in a sterile RNase‐free culture dish on ice with a suitable volume of 1× PBS. Then, the tissue was cut into 0.5 mm^2^ pieces and washed with 1× PBS to remove non‐purpose portions, such as blood stains and fatty layers. Digestion solution was used to dissociate tissues into single cells in 37°C water by shaking for 20 min at 100 rpm. Digestion was terminated with 1× PBS containing 10% fetal bovine serum (FBS) and then filtered using a stacked cell strainer (70‐30 μm). Then, the filtered solution was centrifuged at 300 × g for 5 min at 4°C. The cell pellet was resuspended in 100 μL 1× PBS containing 0.04% BSA. To lyse the residual red blood cells (RBC), 1 mL 1× RBC lysis buffer (MACS 130‐094‐183, 10×) was added to the suspension and incubated on ice for 5 min, followed by centrifuging at 300 × g for 5 min at room temperature. The pellet was resuspended in 100 μL of Dead Cell Removal MicroBeads (MACS 130‐090‐101), and dead cells were discarded using the MACS® Dead Cell Removal Kit (Miltenyi Biotec, 130‐090‐101). Next, the suspension was resuspended in 1× PBS containing 0.04% BSA and centrifuged at 300 × g for 3 min at 4°C. The above steps were repeated twice and the pellet was resuspended in 50 μL of 1× PBS (0.04% BSA). A trypan blue exclusion assay was performed to confirm cell viability. Single‐cell suspensions were counted using a hemocytometer and the concentration was adjusted to 700–1200 cells/μL.

#### 2.1.2 Chromium 10x Genomics library and sequencing

Single‐cell suspensions were loaded onto 10X Chromium using a 10X Genomics Chromium Single‐Cell 3′ kit (V3) following the manufacturer's instructions. cDNA amplification and sequential library construction were performed according to the manufacturer's protocol. Libraries were sequenced on an Illumina NovaSeq 6000 sequencing system (paired‐end multiplexing run, 150 bp) by LC‐Bio Technology Co., Ltd.

### 2.2 Data analysis of single‐cell RNA sequencing

#### 2.2.1 Quality control, UMAP clustering, and marker gene identification

Raw sequencing data were demultiplexed and transformed into FASTQ format using the Illumina bcl2fastq software (version 2.20). The Cell Ranger pipeline (version 3.1.0) was used for barcode processing, 3′‐gene counting, and GRCh38 alignment. Totally, 14,656 single cells were obtained. Dimensional reduction and clustering were performed using Seurat software (version 3.1.1). After quality control, 12,202 remaining cells were used for downstream analysis. The quality control threshold was as follows: (1) The number of genes expressed per cell was limited between 500 and 5000. (2) UMI counts less than 500. (3) Mitochondrial‐DNA derived gene expression was less than 50%. Further data visualization was performed using Uniform Manifold Approximation and Projection (UMAP) after data normalization and principal component analysis (PCA). The FindAllMarkers function was used to identify marker genes for each cluster using the following criteria: (1) genes expressed in more than 10% of cells in a cluster; (2) average log (fold change) of no less than 0.25.

#### 2.2.2 Copy number variation (CNV) inferring

Somatic large‐scale chromosomal CNV score was calculated using “inferCNV” R package. A raw count matrix of scRNA‐seq, annotation file, and gene/chromosome position file was prepared according to data requirements (https://github.com/broadinstitute/inferCNV). T cells from clusters 1/2/15 were used as the normal cell reference. The CNV score was calculated as the quadratic sum of CNV regions.

#### 2.2.3 Pseudo‐time analysis

Developmental pseudo‐time was determined using the Monocle2 software package. The importCDS function was employed to convert the raw counts from the Seurat object to the CellDataSet object. The differentialGeneTest function was employed to select the ordered genes (qval <0.01). Dimensional reduction clustering analysis was carried out using the reduceDimension function, and the order cell function was then used for trajectory inference. Changes in gene expression were tracked over pseudo‐time using the plot_genes_in_pseudotime function.

#### 2.2.4 Scenic analysis

Scenic (Single‐cell regulatory network inference and clustering) was performed using the motifs database for RcisTarget and GRNboost, with SCENIC (version 1.1.2.2), AUCell (version 1.4.1), and RcisTarget (version 1.2.1). Overrepresented transcription factor (TF)‐binding motifs were identified using the gene list in the RcisTarget package. The AUCell package was used to score the activity of each group of regulons in each single cell. The scFunctions (https://github.com/FloWuenne/scFunctions/) package was used to calculate the connection specificity index for all regulons.

### 2.3 Public database acquisition and data analysis

GC bulk RNA‐seq expression data were downloaded from The Cancer Genome Atlas (TCGA, https://portal.gdc.cancer.gov/repository) in the count format. A total of 341 primary tumor samples were included. The count matrix was converted into transcripts per kilobase million (TPM) values. DEG identification and GO and KEGG enrichment analyses were performed using SangerBox 3.0, with default parameters (http://www.sangerbox.com/tool).^18^ Spearman coefficients and *p*‐values between AFP expression and other gene expression were calculated using log_2_(TPM + 1). GC cohorts in the GEO database (GSE15459, GSE34942, GSE57303, and GSE62254) were downloaded for immune infiltration and survival analyses (https://www.ncbi.nlm.nih.gov/geo). A total of 626 patients were enrolled, of whom 617 had prognostic information. The fraction of immune infiltration was analyzed using CIBERSORTx (https://cibersortx.stanford.edu).

### 2.4 In vitro experiment

#### 2.4.1 Immunohistochemical analysis of AFP and DKK1

Formalin‐fixed, paraffin‐embedded tissue sections were deparaffinized and rehydrated. Antigen retrieval was performed in a microwave oven using 10 mM sodium citrate (pH 6.0). For DAB staining, endogenous peroxidase activity was blocked with 0.3% hydrogen peroxide for 10 min and 5% BSA in PBS for 1 h. Slides were then incubated with a primary antibody at 4°C overnight, followed by incubation with an HRP‐linked secondary antibody (HUABIO, Hangzhou, China) at room temperature for 30 min. Diaminobenzidine was used as a chromogen, and the sections were counterstained with hematoxylin.

#### 2.4.2 Cell‐viability assays

FU97 cells (1000/well) were seeded into 96‐well plates and allowed to adhere overnight in complete medium. Four duplicate wells were used for each group. After treatment, cell viability was measured using a CCK‐8 kit (Yeasen Biotechnology), according to the manufacturer's protocol. The incubation time was 1 h. Absorbance was measured at 450 nm using a spectrophotometer.

#### 2.4.3 Wound healing assay

Cells were seeded in six‐well plates at a high density and formed cell monolayers overnight. Then, a line was drawn using a marker on the bottom of the dish, and a sterile 200‐μL pipette tip was used to scratch the cells to generate three separate wounds, moving perpendicular to the line. The cells were gently rinsed twice with PBS and incubated in reduced serum DMEM medium in a humidified 5% CO_2_ incubator at 37°C for 24 h. Images of the scratches were captured using an inverted microscope at 10× magnification at 0 and 24 h of incubation. Each assay was performed in triplicate. The migration distance was calculated using ImageJ software (Version 1.53).

#### 2.4.4 Western blot

FU97 cell lysates were extracted using RIPA Lysis Buffer containing protease and phosphatase inhibitors (Beyotime), fractionated by SDS‐polyacrylamide gel electrophoresis, and electrotransferred to polyvinylidene difluoride (PVDF) membranes. After blocking in 5% skim milk in triethanolamine buffered saline with 1‰ Tween‐20 (TBST) for 1 h at room temperature, the membranes were incubated with primary antibodies (AFP [1:2500, Abcam, EPR20667], GPC3 [1:1000, HUABIO, EM1709‐60], ALB [1:500, HUABIO, ET1702‐55], E‐cadherin [1:10000, Abcam, EP700Y], N‐cadherin [1:5000, Abcam, EPR1791‐4], VIM [1:1000, Abcam, EPR3776], SNAIL [1:1000, Abcam, EPR21043], and GAPDH [1:5000, Abcam, EPR16891]) at 4°C overnight and then with peroxidase‐conjugated secondary antibodies (HRP Conjugated Goat Anti‐Rabbit IgG [1:5000, HUABIO]) for 1 h at room temperature. After washing thrice with TBST, the blot was soaked in Super ECL Detection Reagent (YEASEN) for 10 s. The membranes were then exposed to a film (Kodak) for 10 s in the dark.

#### 2.4.5 RT‐qPCR

Total RNA from FU97 was extracted using the standard RNA simple total RNA extraction method (TIANGEN). First‐strand cDNAs was synthesized from 750 ng total RNA using Hifair® III 1st Strand cDNA Synthesis SuperMix for qPCR (YEASEN) according to the manufacturer's instructions. RT‐qPCR was performed using Hieff® qPCR SYBR Green Master Mix (Low Rox Plus) (YEASEN) on an AB7500 machine. The following primers were used: AFP (forward primer: GGAAGTCTGCTTTGCTGAAGA, reverse primer: CACACCGAATGAAAGACTCGT); GAPDH (forward primer: ACCCACTCCTCCACCTGA, reverse primer: TCCACCACCCTGTTGCTGTA).

### 2.5 Statistical analysis

Data analyses and visualization of single‐cell RNA sequencing were performed using the R software (Version 4.0.1). AddModuleScore, KEGG, and gene set variation analysis (GSVA) were performed to characterize the molecular features of AFPGC. The evolutionary relationship and transcriptional regulation of AFPGC were evaluated using Pseudo‐time and Scenic analyses with correlative R packages. TCGA dataset analyses, including correlation analysis, ROC curve analysis, and KEGG enrichment, were performed mainly using Sangerbox (Vision 3.0). Immune infiltration was detected using online tool CIBERSORTx, and survival analysis was performed using R package “survival.” Consensus clustering was performed with the “ConsensusClusterPlus” package. The experimental data were analyzed and visualized using Prism 8. The migration distance was quantified using ImageJ software (Version 1.53). Statistical significance was set at *p* < 0.05.

## 3 RESULTS

### 3.1 A single‐cell transcriptome atlas for AFPGC

**FIGURE 1:**
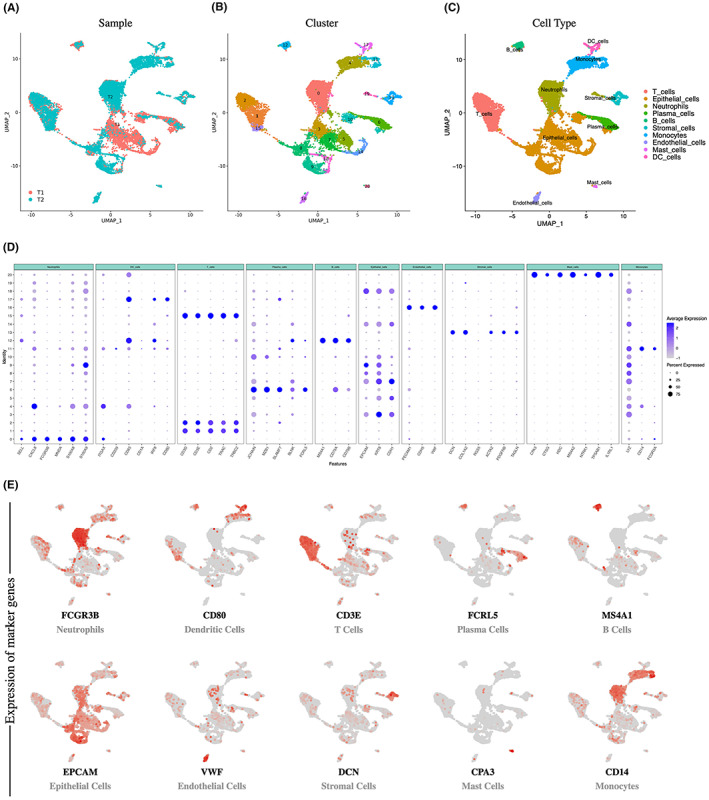
Cellular atlas of AFPGC tumor tissues. (A) Uniform Manifold Approximation and Projection (UMAP) plots for the 12,202 high‐quality cells showing sample origin. (B) UMAP plots showing 21 clusters for the 12,202 cells. (C) UMAP plot showing cell types for the 12,202 cells. (D) Dot plots showing the average expression and percentage of marker genes in 10 cell types. (E) UMAP plots showing expression levels of canonical marker genes for 10 cell types.

To decipher the cell populations and associated molecular characteristics within AFPGC, two AFPGC tumor samples were enrolled in our scRNA‐seq survey (Figure 1A). Two patients were diagnosed with high serum AFP levels (>20 ng/mL); other conditions that may cause an increase in AFP, such as hepatocellular carcinoma, hepatitis, and pregnancy, were excluded. The patients did not receive chemotherapy or radiotherapy prior to surgery. The clinical characteristics of these participants, including serum AFP levels, site of origin, pathological features, and Lauren's classification, are shown in Table 1. After rigid quality control, 12,202 high‐quality cells were retained for downstream analysis (Figure **S1**). After normalization of gene expression and PCA, 21 clusters were identified using graph‐based clustering (Figure 1B). These clusters were assigned to 10 known cell types through marker genes (Figure 1C–E): epithelium (4114 cells, 33.7%, marked with EPCAM, KRT8, and CDH1), neutrophils (2919 cells, 23.9%, marked with FCGR3B and MNDA), T cells (2301 cells, 18.9%, marked with CD3D and CD3E), monocytes (1081 cells, 8.9%, marked with CD14), plasma cells (669 cells, 5.5%, marked with FCRL5), B cells (315 cells, 2.6%, marked with MS4A1), stromal cells (311 cells, 2.5%, marked with DCN and COL1A2), endothelial cells (212 cells, 1.7%, marked with VWF), dendritic cells (202 cells, 1.7%, marked with CD80), and mast cells (78 cells, 0.6%, marked with CPA3) (**Figure** **S2**, **Table** **S1**).

**TABLE 1:** Clinical characteristics of each sample used in scRNA‐seq study and the cell number of each sample after quality control.

Sample	Age	Sex	Histopathological diagnosis	Site of origin	Lauren's classification	Serum AFP (ng/mL)	Number of cells
T1	25	F	Poorly differentiated adenocarcinoma	Posterior wall	Diffuse	83.9	5708
T2	68	M	Moderately poorly differentiated adenocarcinoma	Antrum	Mixed	188.9	6494

### 3.2 Copy number alteration analysis distinguished malignant epithelium from normal gastric epithelium

**FIGURE 2:**
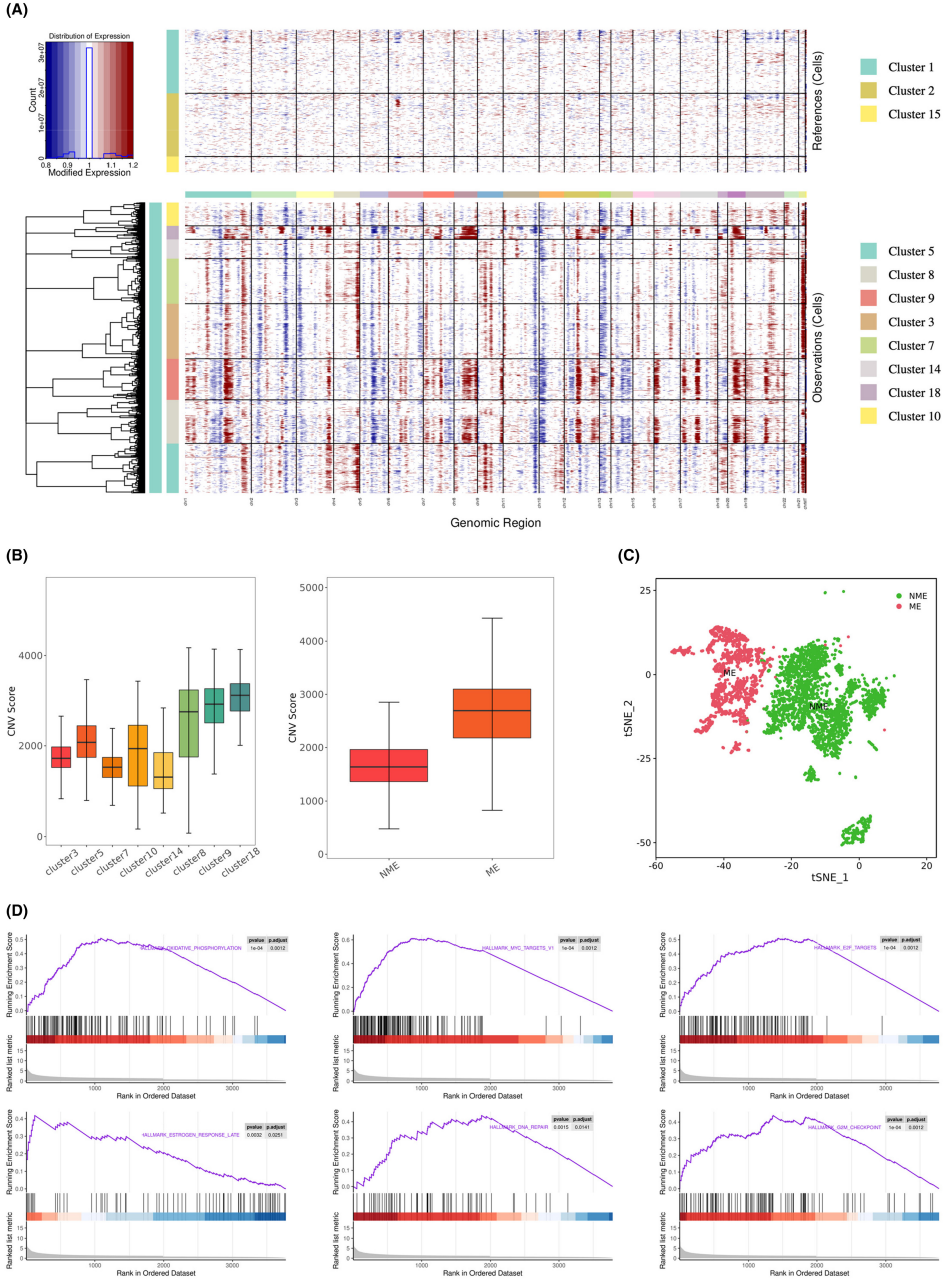
The CNV profile analysis distinguishes malignant epithelium. (A) Heatmap showing large‐scale CNV profile of each epithelial cluster and reference cell subpopulation; the red and blue colors represent high and low CNV levels, respectively. (B) Boxplot on the left side showing the CNV score of each epithelial subpopulation; the other shows the CNV score of malignant epithelium and nonmalignant epithelium. Malignant epithelium: cluster 8/9/18; nonmalignant epithelium: cluster 3/5/7/10/14. (C) t‐Stochastic neighbor embedding (t‐SNE) plots showing fine distinction between ME and NME. (D) Gene set enrichment analysis (GSEA) results showing the enrichment of six ME‐associated gene sets in cluster 8/9/18.

**FIGURE 3:**
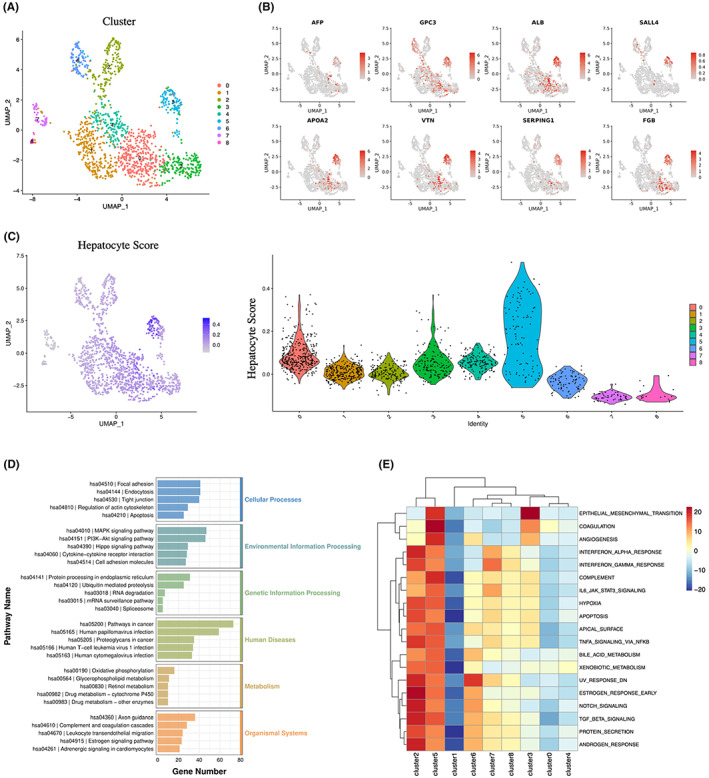
Molecular features of AFPGC at the single‐cell level. (A) UMAP plot showing nine clusters for 1395 malignant cells. (B) Feature plots showing expression of AFPGC‐specific genes AFP and GPC3, and some liver‐specific genes, including ALB, SALL4, APOA2, VTN, SERPING1, and FGB. (C) Feature plot on the left showing hepatocyte score of every single cell. Violin plot showing hepatocyte score of nine clusters. (D) Kyoto encyclopedia of genes and genomes (KEGG) enrichment bar plot for C5 cells. (E) Heatmap showing enrichment scores of hallmark gene sets for nine clusters, based on gene set variation analysis (GSVA).

Eight clusters were identified as epithelial cells, including clusters 3/5/7, 8/9/10, and 14/18. We inferred somatic large‐scale chromosomal CNVs and calculated CNV scores based on a set of reference cell subpopulations (T cells, cluster 1/2/15) through “inferCNV” package (Figure 2A). As illustrated in Figure 2B, clusters 8/9/18 exhibited significantly higher CNV than the reference cells and other epithelial clusters (clusters 3/5/7/10/14) (Figure 2B). Therefore, clusters 8/9/18 and 3/5/7/10/14 were classified as malignant (ME) and nonmalignant (NME) epithelia, respectively. Furthermore, after the extraction of t‐distributed stochastic neighbor embedding (t‐SNE), the malignant epithelium was distinguished from the nonmalignant epithelium (Figure 3C), demonstrating the reliability of the classification.

To decipher the molecular characteristic differences between ME and NME, we performed gene set enrichment analysis (GSEA). Differential expression genes (DEGs) between ME and NME are shown in Table S2. Compared with nonmalignant epithelium, malignant epithelium was enriched in signaling pathways such as oxidative phosphorylation and late estrogen response, with other activated cancer‐related pathways such as MYC target, E2F targets, DNA repair, and G2M checkpoint (Figure 2D).

### 3.3 Molecular features of AFPGC at a single‐cell level

In total, 1395 malignant cells from clusters 8/9/18 were divided into nine major cell subgroups (C0–C8) (Figure 3A). C5 was identified as a typical AFPGC cell with remarkable expression of AFP and GPC3 (Figure 3B). C5 showed prevalent expression of liver‐specific genes, including ALB, SALL4, APOA2, VTN, SERPING1, and FGB (Figure 3B). For a comprehensive look, we scored every malignant cell based on a hepatocyte‐specific gene set from single‐cell research on human liver tissue.^19^ As expected, C5 cells generally had a high hepatocyte score compared to the other clusters, demonstrating their hepatoid differentiation characteristics (Figure 3C). DEGs between C5 and other malignant cells were identified and sequential KEGG analysis was performed. C5 cells, also known as AFPGC cells, were enriched for metabolic pathways such as oxidative phosphorylation and drug metabolism. In addition, abnormal activation of signaling pathways such as MAPK and Hippo may be related to the malignant phenotype of AFPGC (Figure 3D). The enriched pathways for C5 are listed in Table S3.

In addition, we explored hallmark scores based on GSVA. AFP‐producing cells showed significant enrichment for coagulation and complement terms, which were also specifically activated in liver tissue (Figure 3E). Other enriched gene sets are crucial for cancer progressions, such as EMT and angiogenesis, providing a reasonable explanation for their high propensity for liver and lymph node metastasis (Figure 3E).

**FIGURE 4:**
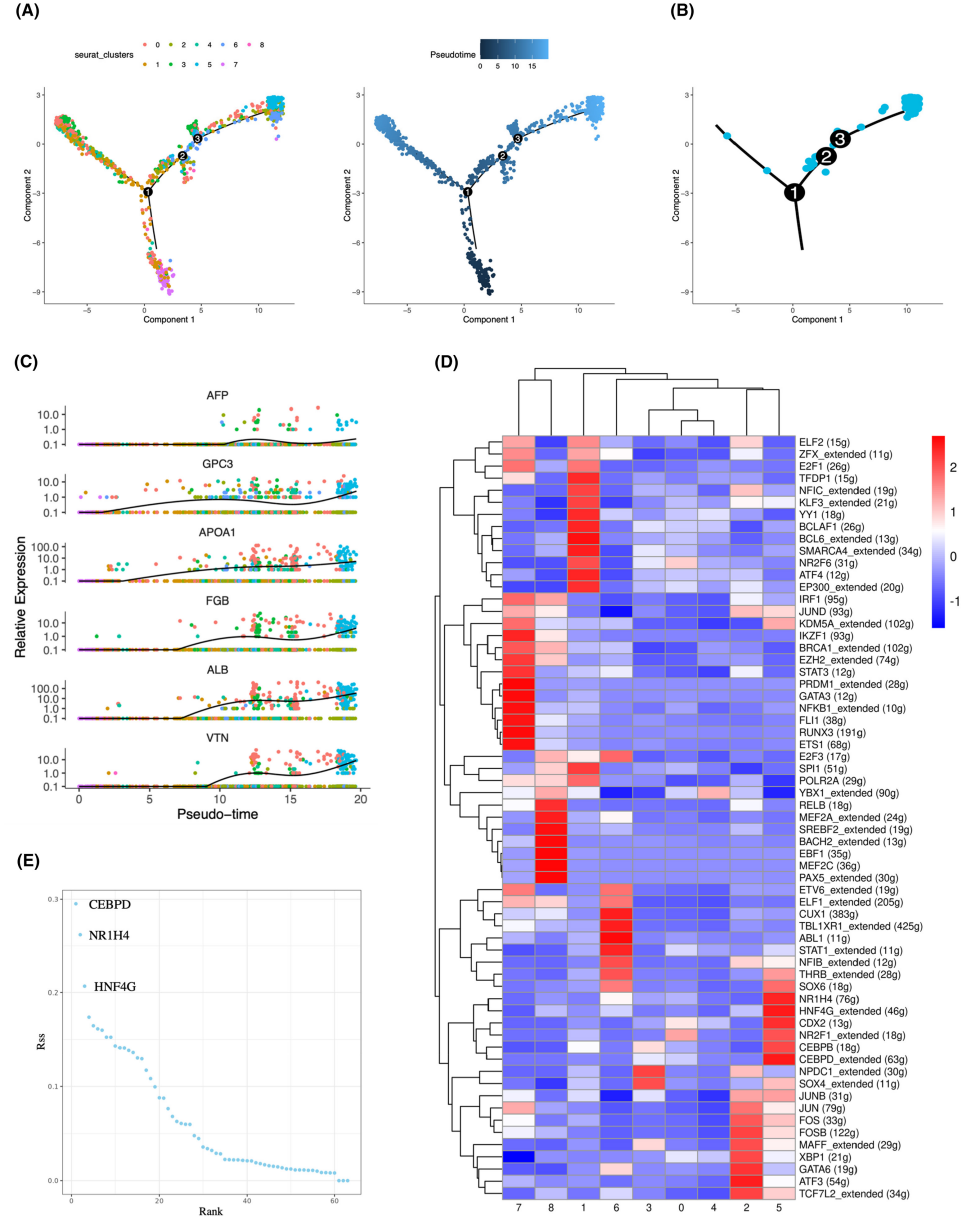
Pseudo‐time analysis and Scenic analysis of AFPGC subpopulation. (A) Monocle 2 pseudo‐time analysis for nine malignant clusters. (B) The trajectory of C5 cells constructed by Monocle 2. Each point corresponds to a single cell. (C) Gene expression distribution map based on evolution state for AFP, GPC3, APOA1, FGB, ALB, and VTN. (D) Heatmap displays the activity of different regulons across nine malignant clusters. (E) The scatter diagram highlights three transcription factors with the highest regulation activity in AFPGC cells. RSS, Regulons score.

Cell trajectory analysis of the malignant epithelium was performed to further investigate the evolutionary pattern of AFPGC (Figure 4A). First, results showed that AFPGC evolved from a small number of malignant cells with hepatoid differentiation potential but low AFP expression (Figure 4B). In the middle and late stages of evolution, these cells were translated into typical AFPGC cells with high expression of AFP and GPC3, accompanied by significant hepatoid differentiation features, manifested as synchronous changes in liver‐specific genes, such as APOA1, FGB, ALB, and VTN (Figure 4C).

To elucidate the transcriptional regulation pattern of AFPGC, Scenic analysis was performed (Figure 4D). CEBPD displayed the highest regulatory activity in AFPGC cells, followed by NR1H4 and HNF4G (Figure 4E). CEBPD has been demonstrated to promote growth, metastasis, and drug resistance in multiple tumor types and can serve as a potential target for cancer treatment.^20^ Other significantly enriched TFs, such as NR1H4, THRB, and JUN, were reported to participate in the development process and maintain normal physiological functions in the liver.^21^, ^22^, ^23^

### 3.4 DKK1 promotes AFP expression in AFPGC

**FIGURE 5:**
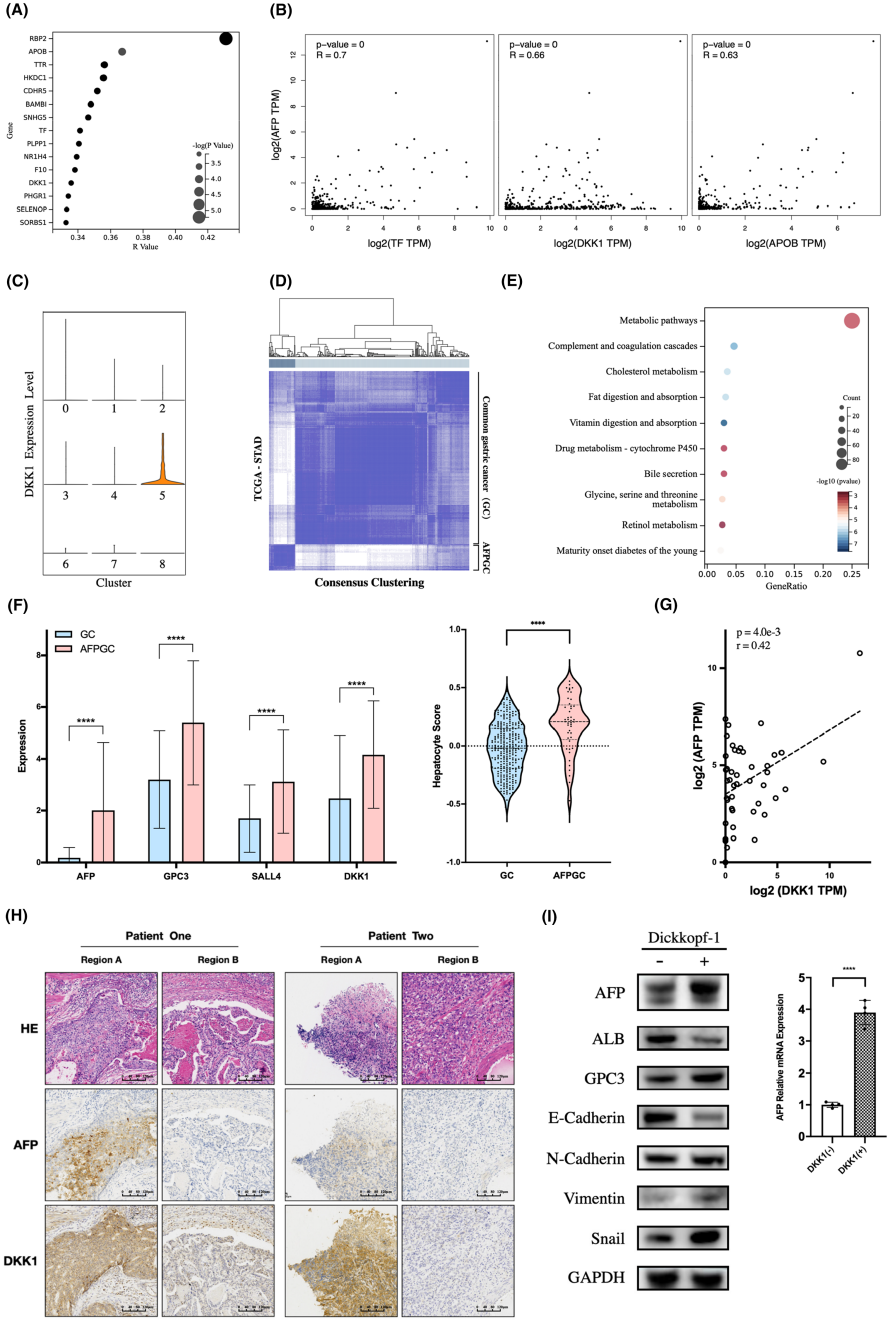
DKK1 expression is related to AFP in AFPGC. (A) Bubble chart showing the top 15 genes related to AFP expression via correlation analysis. (B) Scatter plots showing the correlation between AFP expression and three selected genes using TCGA gastric cancer dataset. (C) Violin plot shows DKK1 expression level among malignant epithelium clusters. (D) Consensus clustering divided 341 cases (from TCGA‐STAD cohort) into two groups, namely the AFPGC group (45 cases) and common gastric cancer (GC) group (296 cases). (E) Bubble chart showing the enriched pathway of AFPGC upregulated genes. (F) Bar diagram showing the expression level of several marker genes and hepatocyte score based on GSVA. (G) Correlation between AFP and DKK1 expression among 45 AFPGC patients in TCGA cohort. (H) HE stains and immunohistochemistry on another two AFPGC tissues. All pictures were captured at a magnification of 100×. (I) Western blot showing AFP, ALB, GPC3, and several EMT marker expression in FU97 cells with or without DKK1 stimulation. RT‐qPCR was performed to detect AFP transcription level. FU97 was treated with DKK1 (0.1 μg/mL) and incubated for 48 h before extracting protein and total RNA.

To investigate the mechanism of AFP expression regulation, we first screened the genes associated with AFP expression in AFP‐producing cells. The results of correlation analysis are presented in Table S4. Among the top 15 related genes, most were highly expressed in human liver tissues, including APOB, TTR, TF, and F10 (Figure 5A). We subsequently validated these 15 genes using transcriptome data of GC in TCGA database and found that three (TF, APOB, and DKK1) were significantly correlated with AFP expression (*p* < 0.01, *R* > 0.6) (Figure 5B). Previous studies have shown that TF and APOB are highly expressed in human liver tissues, where they exert normal physiological functions in liver cells.^24^, ^25^ Generally, they are considered to be products of hepatic differentiation, such as AFP. However, their relationship with tumor malignancy remains poorly understood.

DKK1 is physiologically expressed in the bladder and placenta and is involved in human embryonic development and bone formation.^6^ DKK1 is an inhibitor of the WNT signaling pathway, which is upregulated in some cancer types and is involved in tumor proliferation, invasion, and immunosuppression.^9^, ^26^ DKK1 was specifically expressed in Cluster 5 of the malignant epithelium **(**Figure 5C **)**. Therefore, we sought a deeper insight into DKK1.

To accurately identify patients with AFPGC in the TCGA GC dataset, we performed consensus clustering using the top five marker genes of C5, including APOA2, ALB, TSHZ2, EXT1, and DEFB1 (Table S5). A total of 341 patients in the TCGA‐STAD dataset were divided into an AFPGC group (45 cases) and a common GC group (296 cases) (Figure 5D). GO enrichment analysis showed that AFPGC upregulated metabolism‐related, complement, coagulation, and drug metabolism pathways (Figure 5E), with significantly higher expression of AFP, GPC3, and SALL4 (Figure 5F). The hepatocyte score of AFPGC was significantly higher than that of common GCs. The results above indicate that the molecular characteristics of the AFPGC group in the TCGA‐STAD dataset were extremely similar to those of the C5 subpopulation and also demonstrated the reliability of consensus clustering. DKK1 expression in AFPGC was significantly higher than that in common GC and positively correlated with AFP (*R* = 0.42, *p* < 0.01) (Figure 5F,G). Immunohistochemical analysis of two other AFPGC tissues showed that DKK1 was expressed in AFP‐positive areas, whereas it was rarely expressed in AFP‐negative areas (Figure 5H).

In vitro, FU97, an AFPGC cell line,^27^ was treated with DKK1 and showed a significant increase in the transcription and protein levels of AFP (Figure 5K), providing strong evidence that DKK1 expression is associated with AFP in AFPGC and may be involved in the regulation of AFP expression.

### 3.5 DKK1 promotes malignant phenotype of AFPGC

**FIGURE 6:**
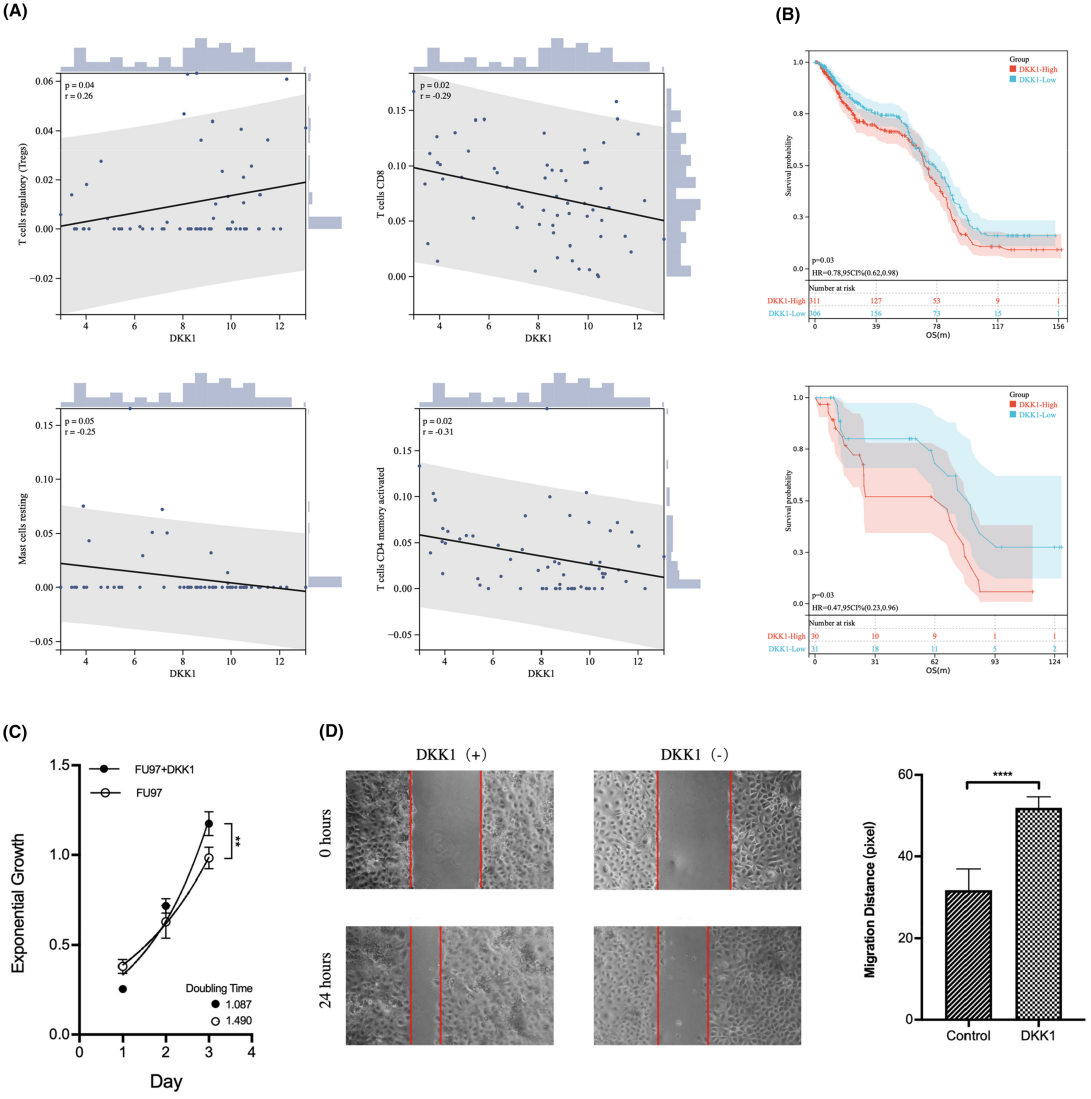
DKK1 has effects on the malignant phenotype of AFPGC. (A) Correlation analysis between DKK1 and immune cell infiltration in AFPGC. (B) Gastric patients in the GEO cohort were divided into two groups according to DKK1 expression level, and the survival rate of these two groups indicated that the gastric patients with higher DKK1 expression levels had worse OS (*p* < 0.05). Among them, 61 AFPGC patients were identified according to consensus clustering, and survival analysis confirmed the poor prognosis of DKK1‐high AFPGC (*p* < 0.05). (C) CCK‐8 assay was performed to compare proliferation levels with and without DKK1 treatment. (D) Wound‐healing assay was performed to assess the effect of DKK1 on AFPGC cell migration. FU97 was treated with DKK1 (0.1 μg/mL) and incubated for 24 h before recording. Images were captured at a magnification of 100×.

Next, we investigated the effect of DKK1 on the malignant phenotype. To obtain a larger sample size for reliable AFPGC results, we enrolled 626 GC patients from the GEO database, and 617 of them had prognostic information. The combination and normalization details of the GEO database are presented in Figure S2. Among them, 61 patients were identified as having AFPGC, based on consensus clustering. The fraction of immune cell infiltration was detected and correlation analysis was performed. DKK1 expression in GC positively correlated with regulatory T cell (Treg) infiltration and negatively correlated with CD8^+^ T cells, resting mast cells, and activated memory CD4^+^ T cells (Figure 6A), indicating that DKK1 is associated with immunosuppression in GC. Furthermore, survival analysis showed that GC patients with high DKK1 expression had worse overall survival than patients with low DKK1 expression (Figure 6B). This phenomenon was also observed in AFPGC. Sequential in vitro experiments were performed to verify the effect of DKK1 on AFPGC. When stimulated by DKK1, FU97 cells showed an accelerated proliferation level with a shortened doubling time in the CCK8 assay (Figure 6C). In addition, compared with the control group, the invasive ability of FU97 cells was significantly enhanced after stimulation with DKK1, as confirmed by the wound healing assay (Figure 6D). Furthermore, DKK1 enhanced the epithelial–mesenchymal transition (EMT) level of AFPGC, as demonstrated by the upregulation of N‐cadherin, vimentin, and snail and downregulation of E‐cadherin expression (Figure 5I). Taken together, in vitro experiments suggest that DKK1 may mediate the malignant phenotype and lead to poor prognosis of AFPGC.

## 4 DISCUSSION

Bourreille et al. first reported and introduced the concept of AFPGC in 1970.^28^ Since then, this particular type of GC has received increasing attention from clinicians and researchers owing to its highly malignant behavior and poor prognosis. However, owing to the complexity and rarity of the disease, research has been slow. In addition, there is still a lack of effective treatments. Therefore, it is imperative to investigate the mechanism of AFPGC development and explore potential therapeutic targets.

Next‐generation sequencing (NGS) is a powerful tool for exploring the molecular pathogenesis of malignant tumors and guiding cancer diagnosis and treatment. Few recent studies have performed WES on AFPGC tissues^3^, ^4^; however, little is known about the pathogenesis of AFPGC and the causes responsible for its malignant biological behavior. Some studies have implied that the underlying mechanism of pathogenesis might be relevant to lineage reprogramming and hepatocyte‐like differentiation, according to observations and analyses of several clinical samples.^1^, ^16^, ^17^ Therefore, in this study, we used an alternative approach of performing single‐cell transcriptome sequencing on AFPGC tumor tissues based on the following considerations. First, the dominant view is that AFPGC evolved from common GC,^29^ and single‐cell sequencing has a greater advantage in revealing intercellular transformation relationships and tumor evolution.^30^ In addition, several researchers have performed WES on AFPGC, but no studies have reported its molecular characteristics at the single‐cell level. Additionally, according to clinical retrospective analyses and case studies, intra‐tumor heterogeneity within AFPGC is complex. In fact, only a few patients showed AFP‐positivity by immunohistochemistry,^2^ probably because AFPGC cells constitute only a small fraction of the entire tumor tissue. Through single‐cell sequencing, the heterogeneity of tumors can be analyzed at an unprecedented resolution.

We identified a typical subpopulation of AFPGC cells using malignant cell subclustering and marker genes. Similar to previous findings, the AFP‐producing adenocarcinoma fraction represented only a small fraction of the entire tumor tissue, in which case, traditional bulk sequencing may not be an ideal fit. Previous studies have shown that hepatoid adenocarcinoma is the most common pathological type of AFPGC,^2^ characterized by hepatocyte‐like morphology and the expression of hepatocellular carcinoma marker genes, such as AFP, GPC3, and SALL4.^31^, ^32^ Thus, we scored each tumor cell using GSVA enrichment based on hepatocellular marker genes, demonstrating the similarity between the AFPGC subpopulation and hepatocytes in the expression profile. In addition, the KEGG enrichment analysis showed that AFPGC has many physiological functions similar to hepatocytes, such as drug metabolism, complement, and coagulation factor synthesis. This suggests that the idea of drawing on studies related to human liver development contributes to our understanding of the pathogenesis of AFPGC. Leaving aside the traditional mining of AFPGC mutations, we have looked at the characteristics of this rare disease from a completely new perspective, and for the first time, the molecular function of AFPGC has been characterized at the single‐cell level.

The AFPGC subpopulation displays high regulatory activity of several transcriptional factors such as CEBPD, NR1H4, and THRB. CEBPD is a leucine zipper transcription factor that can promote malignant biological behavior in various cancer types.^33^, ^34^ This suggests that targeted therapy for CEBPD may be beneficial in improving the prognosis of AFPGC. Other enriched TFs, such as NR1H4, encoding the farnesoid X receptor (FXR), control bile acid homeostasis in liver tissue.^21^ The similarity between AFPGC cells and hepatocytes in transcriptional regulatory patterns suggests that AFPGC can mimic the developmental processes and physiological functions of hepatocytes. Further pseudo‐time analysis suggested that the evolution of AFPGC was accompanied by hepatoid differentiation, showing simultaneous upregulation of hepatocyte‐related genes. The dynamic changes in AFP expression with tumor evolution and the different compositions of AFP‐producing adenocarcinoma cells in each period can partly explain the clinical mismatch between serum AFP levels and immunohistochemical properties. In addition, we found activated signaling pathways, such as MAPK and HIPPO, by enrichment analysis of the AFPGC subpopulation, which may be potentially effective targets for treatment. Meanwhile, EMT and angiogenesis were increased in the AFPGC subpopulation compared with their level in the common GC subpopulation, mediating early metastasis and poor prognosis in clinical practice.

In addition to the pathogenesis and molecular features, exploring AFP expression regulation and malignant phenotypic mechanisms is an important part of AFPGC research. Kataoka et al.^35^ and Cho et al.^36^ found that ATBF1 mutations may mediate AFP expression and malignancy in GC. Liu et al. discovered that MUC19 could upregulate AFP expression in GC cells and promote the development of hepatoid adenocarcinoma of the stomach (HAS).^37^ In this study, we found that DKK1 was associated with AFP expression in AFPGC by combining single‐cell and TCGA transcriptomic data, which was further confirmed by a DKK1 stimulation assay and immunohistochemistry of two other AFPGC patient slides. Interestingly, GPC3 was also upregulated by DKK1 in vitro, accompanied by a decrease in ALB expression. Normally, GPC3 appears in liver cancer and fetal liver, whereas ALB is specifically expressed in the adult liver.^38^, ^39^ Based on the dynamic changes in the protein profile during liver differentiation,^40^ DKK1 stimulation may reverse AFPGC differentiation. However, the specific mechanism requires further investigation.

Previous studies have shown that DKK1 promotes malignant phenotypes in hepatocellular carcinoma, including tumor angiogenesis, invasion, and metastasis. It has recently been demonstrated that DKK1 promotes immune escape and impairs anti‐PD‐1 therapeutic efficacy in GC.^15^ Therefore, we further explored the effect of DKK1 on the malignant phenotype of AFPGC and found that it promoted AFP expression, as well as proliferation, migration, and EMT of AFPGC. From another point of view, a previous study demonstrated that AFP could promote malignant biological behaviors of AFPGC.^41^ Blocking DKK1 expression could possibly release the malignancy‐promotional role of AFP secretion. Therefore, DKK1 may be a potential target for anti‐AFPGC therapy. Clinical trials have been conducted combining DKN‐01, an antibody to DKK1, with tislelizumab and chemotherapy as first‐line treatments for patients with GC and gastroesophageal junction cancer, with a preliminary objective response rate (ORR) of 68% and disease control rate of 100%. In particular, patients with high DKK1 expression achieved an ORR of 100%.^42^ However, the efficacy of anti‐DKK1 treatment in AFPGC requires further validation. In conclusion, we analyzed and characterized the molecular characteristics of AFPGC at the single‐cell level. We also demonstrated functional, developmental, and transcriptional similarities between AFPGC cells and hepatocytes, which will contribute to our understanding and further study of this complex disease. We also revealed the involvement of DKK1 in AFP expression regulation and promotion of malignant phenotype by combining scRNA‐seq with TCGA and GEO cohorts, and finally verified it through a series of in vitro experiments. Therefore, DKK1 may serve as a novel diagnostic marker and therapeutic target for AFPGC.

This study has several limitations. First, the number of samples enrolled in single‐cell RNA sequencing was limited. However, AFPGC is a rare tumor; therefore, acquiring sufficient samples is difficult. In addition, most patients underwent chemotherapy rather than surgery. Additionally, the promoting effect of DKK1 on malignant tumor phenotypes needs to be further verified in vivo as well as the effectiveness of anti‐DKK1 therapy against AFPGC.

## AUTHOR CONTRIBUTIONS

**YANXIA MEI:** Conceptualization (lead); data curation (lead); formal analysis (lead); software (lead); validation (lead); writing – original draft (lead); writing – review and editing (lead). **Ming Li:** Data curation (equal); formal analysis (equal); writing – original draft (equal); writing – review and editing (equal). **Jihang Wen:** Formal analysis (equal); writing – original draft (equal); writing – review and editing (equal). **Xiang‐Xing Kong:** Formal analysis (equal); funding acquisition (equal); software (equal). **Jun Li:** Conceptualization (equal); funding acquisition (equal); project administration (lead); resources (lead); supervision (lead); writing – review and editing (equal).

## FUNDING INFORMATION

This research was supported by the National Natural Science Foundation of China (grant nos. 82,103,684, 11,932,017, and 82,172,851), Huadong Medicine Joint Funds of the Zhejiang Provincial Natural Science Foundation of China (grant no. LHDMY22C060002). The authors have no other relevant affiliations or financial involvement with any organization or entity with a financial interest in or financial conflict with the subject matter or materials discussed in the manuscript apart from those disclosed.

## ETHICS STATEMENT

The study was approved by the Ethics Committee of The Second Affiliated Hospital, Zhejiang University School of Medicine, Hangzhou. The authors state that they have obtained appropriate institutional review board approval or have followed the principles outlined in the Declaration of Helsinki for all human or animal experimental investigations. In addition, for investigations involving human subjects, informed consent has been obtained from the participants involved.

## Supporting information


Supplementary Figure S1.

Supplementary Table S1.

Supplementary Table S2.

Supplementary Table S3.

Supplementary Table S4.

Supplementary Table S5.

## Data Availability

The expression matrix of single‐cell RNA sequencing reported in this paper have been deposited in the OMIX, under accession numbers OMIX002217. And Publicly available datasets were analyzed in this study. This data can be found here: TCGA (https://portal.gdc.cancer.gov/) and GEO (https://www.ncbi.nlm.nih.gov/geo/).
