# Supplementary material for: Single‐cell characteristics and malignancy regulation of alpha‐fetoprotein‐producing gastric cancer
Source: Cancer Med. 2023 Apr 5;12(10):12018–33. doi: 10.1002/cam4.5883 (PMC10242870; doi:10.1002/cam4.5883)
Supplement: Supplementary file 1 — Supplementary Figure S1. [file CAM4-12-12018-s002.docx]

Supplementary Material

# Supplementary Figures and Tables

A B


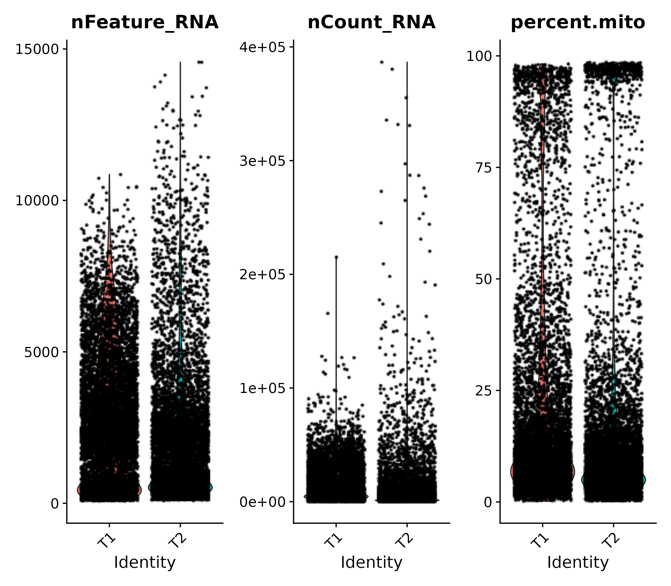

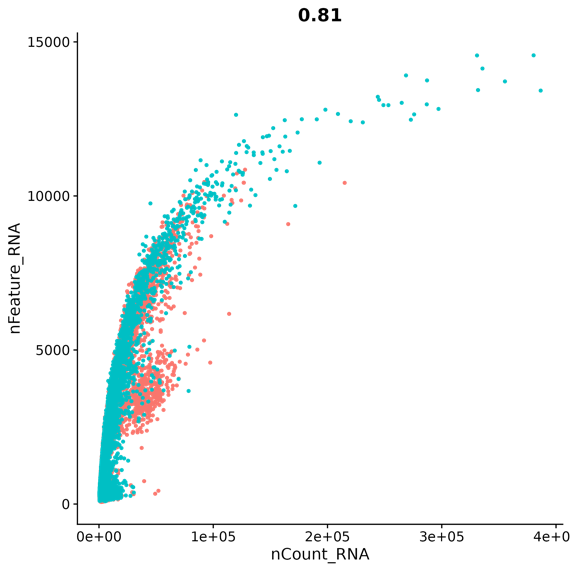


C D


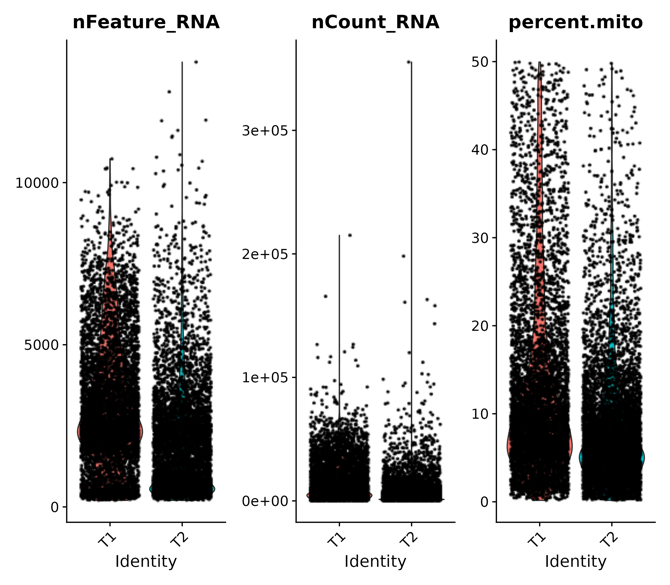

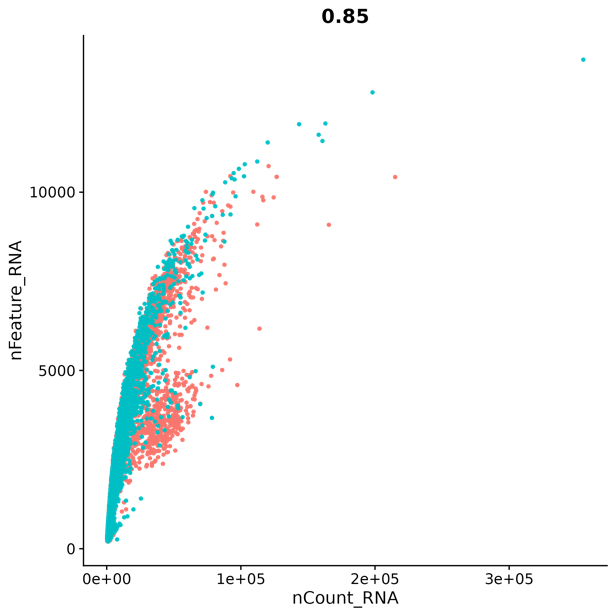


**Supplementary Figure S1** Quality control of two AFPGC samples. (A, C) Violin and dot plots showing number of genes (nCount_RNA), transcripts (nFeature_RNA) and percentage of mitochondrial gene (percent.mito) between two AFPGC tissue cells before and after quality control. (B, D) Scatter plot showing the number of genes and the number of transcripts detected in each cell before and after quality control.


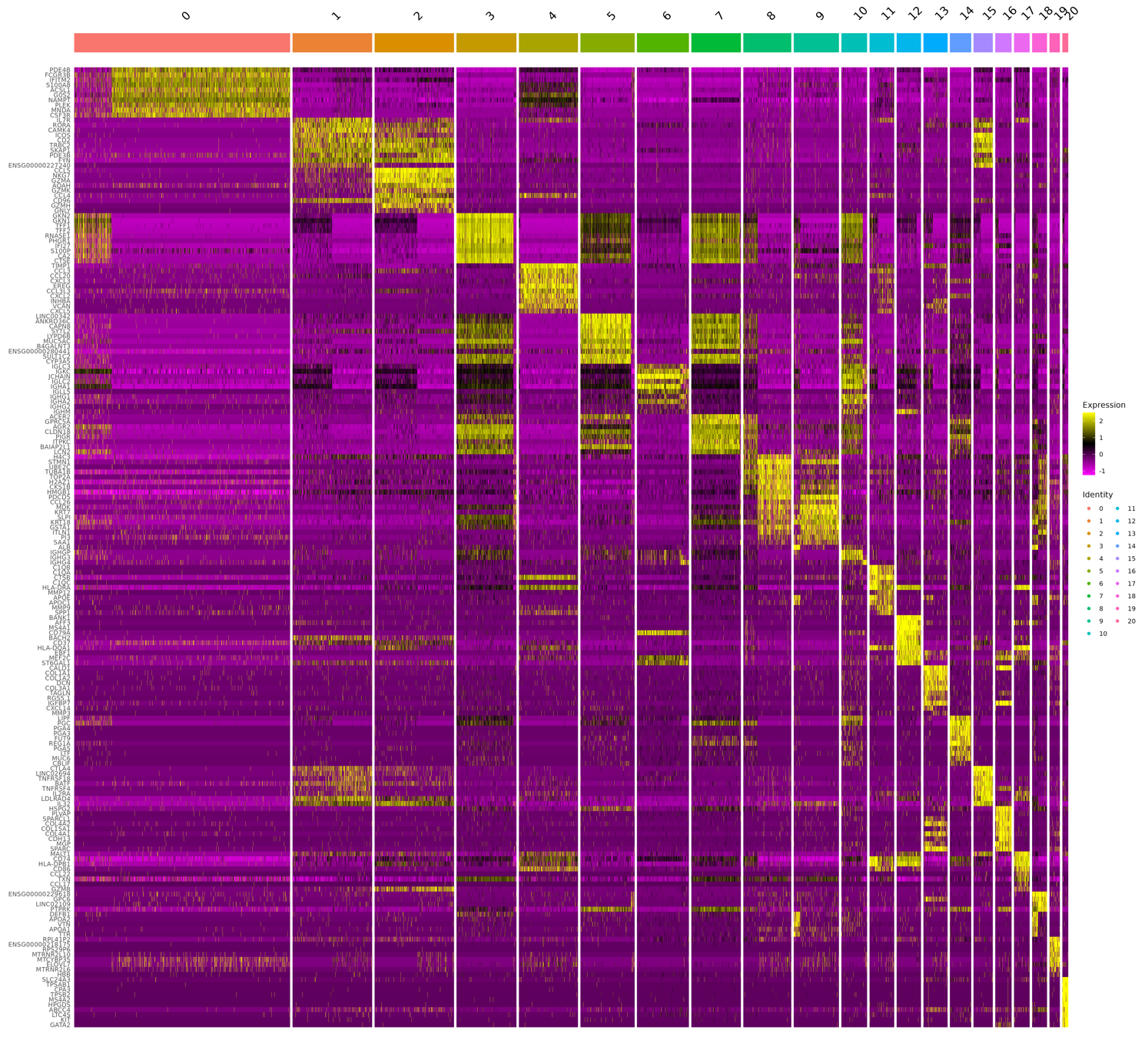


**Supplementary Figure S1** Heatmap showing top ten marker genes of 21 clusters

# Supplementary Data

**Supplementary Table S1** All marker genes of 21 clusters. These selects markers genes are expressed in more than 10% of the cells in a cluster and average log (Fold Change) of greater than 0.25

**Supplementary Table S2** Differential expression genes (DEGs) between malignant epithelium and non-malignant epithelium

**Supplementary Table S3** All enriched pathways of C5 subpopulation cells by KEGG analysis

**Supplementary Table S4** Correlation analysis between AFP expression and other genes in C5 subpopulation

**Supplementary Table S5** All Marker genes of Cluster 5 subpopulation
